# Association between anemia, physical performance and cognitive function in Iranian elderly people: evidence from Bushehr Elderly Health (BEH) program

**DOI:** 10.1186/s12877-021-02285-9

**Published:** 2021-05-24

**Authors:** Maryam Marzban, Iraj Nabipour, Akram Farhadi, Afshin Ostovar, Bagher Larijani, Amir Hossein Darabi, Elnaz Shabankari, Mohamad Gholizade

**Affiliations:** 1 Clinical Research Development Center, The Persian Gulf Martyrs, Bushehr University of Medical Sciences, Bushehr, Iran; 2 The Persian Gulf Tropical Medicine Research Center, The Persian Gulf Biomedical Sciences Research Institute, Bushehr University of Medical Sciences, Bushehr, Iran; 3 The Persian Gulf Marine Biotechnology Research Center, The Persian Gulf Biomedical Sciences Research Institute, Bushehr University of Medical Sciences, Bushehr, Iran; 4 Osteoporosis Research Center, Endocrinology and Metabolism Clinical Sciences Institute, Tehran University of Medical Sciences, Tehran, Iran; 5 Endocrinology and Metabolism Research Center, Endocrinology and Metabolism Clinical Sciences Institute, Tehran University of Medical Sciences, Tehran, Iran; 6 Department of Nutrition Science, Bushehr University of Medical Sciences, Bushehr, Iran

**Keywords:** Hemoglobin, Cognition, Anemia, Elderly, Physical, Walking speed, Handgrip

## Abstract

**Background and objectives:**

The present study aimed to investigate the relation between anemia and hemoglobin (Hgb) concentration, physical performance, and cognitive function in a large sample of Iranian elderly population**.**

**Methods:**

Data were collected from Bushehr elderly health (BEH) program. A total of 3000 persons aged ≥60 years were selected through multistage random sampling. Hemoglobin values lower than 12 and 13 g/dL were considered as anemia for women and men, respectively. The cognitive function was measured using the Mini-cog test and Category fluency test (CFT), and the physical function was measured using handgrip strength (muscle strength), Relative handgrip strength (RHGS), and 4.57-m usual gait speed. Univariate and adjusted multivariate logistic regression and linear regression with Stata MP (version 15) were run, and a *p*-value of < 0.05 was used as statistically significant for all analyses.

**Results:**

Among participants, 7.43% were anemic, and 115 (51.57%) simultaneously had anemia and cognitive disorder. There were significant associations between red blood cell count (RBC), hemoglobin (Hgb), platelet count (PLT), and hematocrit percentage (HCT) with cognitive impairment. Additionally, Hgb concentration was significantly associated with all physical measures (Mean handgrip, Relative handgrip, and usual gait speed) and late recall (mini-cog) among the whole participants. This association remained statistically significant after considering multi-cofounders. In contrast, after stratifying the participants by gender, the association between Hgb concentration and usual gait speed was decreased in both men and women; moreover, Hgb association with cognitive measures (category fluency test and late recall) was no longer significant (all *p*-values > 0.05).

**Conclusion:**

There was a cross-sectional and significant association between anemia and functional variables (e.g., Relative and mean handgrip) in Iranian elderly population, whereas Semantic memory, Late recall, and walking were more affected by gender.

## Background

Anemia is a considerable global problem which is defined through various criteria in older adults. The most acceptable definition is presented by the World Health Organization (WHO), which is defined as a hemoglobin (Hgb) concentration of lower than 13 g/dl (> 13 g/dl) for men and lower than 12 g/dl (> 12 g/dl) for women [1]. The prevalence of anemia is more than 10 to 24% in older ages [2, 3], which reaches 48% in hospitalized older individuals [4], and it affects 67% of senior adults living in a nursing home [5]. Although anemia is not considered a disease entity, it may have some adverse effects on health and could be associated with several clinical complications like reduced muscle strength, physical performance, cognitive impairment, and dementia [6–8].

According to the World Population Prospects, the number of older adults over 65 is expected to rise from 703 million in 2019 to 1.5 billion in 2050. The growth rate has dramatically increased compared to the previous predictions. The advanced age population also keeps on rising in lower- and middle-income countries such as Iran [9]. Iran is experiencing a rapidly growing rate of the elderly. It is expected that the elderly population will increase from 6.17% in 2015 to 21.7% in 2050 [10]. Aging is associated with functional disabilities (physical and cognitive) and age-related diseases, which result in a significant increase in the therapeutic costs and influences in socioeconomic status [9]. Cognitive impairment (CI) is a common complication of aging increasing, especially in developing countries. Studies demonstrate that 50 million people in the world currently have dementia, and it will increase to 131 million by 2050 [11, 12]. The most massive increases are expected in the countries with the highest rates of anemia [11, 13].

Potential cognitive impacts related to inadequate iron might stem from cerebral hypoxia [7, 14], insufficient neurotransmitter synthesis, or low myelin integrity [6]. Based on the results of the studies, the causal relationship between low Hgb and adverse outcomes such as impaired physical and cognitive performance is unclear [7, 8, 13, 15]. No trials have confirmed the complete improvement of cognitive and physical function after the treatment of anemia in the elderly [13, 15]. Although some studies have confirmed the significant relationship between Hgb concentration and CI [6, 8], others have exhibited no significant association between anemia and CI [13, 16].

With the growing number of demented elderly and lack of an effective pharmacological cure for this disease, the most crucial preventive strategy is controlling its modifiable risk factors such as anemia, but this preventive approach is very complicated [8, 12]. To address the fundamental questions about anemia related to cognitive impairment in older adults and its complex consequences, it will be critical for ensuring commensurate research, clinical, and public health responses to anemia. Therefore, the present study aims to investigate the relation between Hgb concentration, physical performance, and cognitive function in the elderly.

## Methods and measurements

### Participants and data sources

The BEH Program is a population-based, prospective cohort study currently being conducted in Bushehr, Iran. The target population of this study was all men and women aged 60 years and over living in Bushehr, the center of a southern province of Iran in the north of the Persian Gulf. The methodology of the BEH program has been previously described elsewhere [17, 18]. A total of 3000 persons aged ≥60 years were selected through a multistage, stratified cluster random sampling method from an estimated population of about 10,000 individuals (Based on Bushehr health center information). The number of participants was proportional to the number of households residing in each of the 75 strata of Bushehr port, Iran. Baseline measurements of the first stage were implemented from March 2013 to October 2014. The second stage began in October 2015, and data was recollected for 2 years. The inclusion criteria were as follows: age more than or equal to 60 years, comprising both sexes (males and females), residency in Bushehr port since at least 1year before the recruitment and having no plan to leave the city for the next 2 years, adequate physical and mental ability to participate in the evaluation program and signing a written informed consent. From among 3297 participants who met the inclusion criteria, 3000 participants (1455 men and 1545 women) accepted to be involved (participation rate: 91.0%). Of those 3000 people who participated in the first phase, 2426 people remained in the second phase of the study (80% response rate), and 574 people were excluded due to death, migration, or unwillingness to participate in the study. They were checked by a trained nurse once a year for the outcomes, and a form was given to the participants for self-reporting at the earliest opportunity after the incident of any of the marked outcomes. All participants were asked to fill validated questionnaires that were translated into Persian. All Bushehr Elderly Health program participants provided written informed consent, and the Research Ethics Committee approved the study protocol of Bushehr University of Medical Sciences (Reference number: B-91–14-2).

### Laboratory examinations

All participants were asked to provide 10 mL of the whole blood taken by a trained nurse after 8–12 h of fasting for laboratory tests. The whole blood included complete blood count (CBC), Mean corpuscular volume (MCV), Red blood cell count (RBC), Hgb, White blood cells (WBC), Platelets (PLT), Red Cell Distribution Width (RDW), Mean corpuscular hemoglobin (MCH), Fasting blood sugar and lipid profile. The automated hematology analyzer, Medonic CA620 (Menarini Diagnostic Srl, Florence, Italy), was used for the measurements.

According to the World Health Organization criteria, anemia is defined as Hgb concentrations lower than 13 g/dl for men and lower than 12 g/dl for women [1].. Thus, the anemic cases were categorized by mean corpuscular volume (MCV) and Hgb concentration. Microcytic anemia was defined as MCV lower than 80 femtolitre MCV < 80, and normocytic anemia MCV 80 to 100 femtolitre and macrocyte anemia were define as MCV > 100 femtolitres [9].

### Measures of cognitive function

Evaluation of cognitive function started in the second phase of the study. Mini-cog and category fluency test (CFT) were used for evaluating the cognitive function, which have already been validated and translated for use in primary care in Iran [19]. Men and women who had a low score in one or more tests were considered cognitively impaired.

**Mini-Cog** is a validated and brief screening test for measuring CI that takes approximately 3 min to perform and consists of two parts; the first part assesses the participant’s ability to recall three words. Individuals who could recall all three words are considered to have normal cognition abilities and others as cognitively impaired. For those who could only recall one or two words, a clock-drawing test is performed. Those who could correctly draw the clock are assumed to have a normal cognitive function and others to have an impaired cognitive function [17]. This test has the least language content; therefore, lower cultural and educational bias is found in this test [20].

**Categorical verbal fluency test (CFT)**: It is a short screening test that evaluates cognitive function (semantic memory). This CFT requires the participant to name as many examples of the category “animal” as possible within 1 min. A previous report has shown that a cut-off score of 13/14 on the CFT was able to distinguish patients with AD from control subjects with a sensitivity of 0.91 and a specificity of 0.81 [21].

### Measures of physical function

To test physical function, handgrip strength (muscle strength), Relative Handgrip Strength (RHGS) [22], and 4.57-m usual gait speed (physical performance) were measured. The intensity of the physical activity level in 24 h of work, sports, and leisure time was expressed in metabolic equivalents. Four categories were defined based on the level of physical activity (sedentary: 1–1.39, low active: 1.4–1.59, active: 1.6–1.89, very active: 1.9–2.5) [23]. This instrument is a valid self-report questionnaire that has been validated among Iranian adolescents for Farsi language [23, 24].

### Relative handgrip strength (RHGS)

Recently, using BMI to adjust for handgrip strength has been recommended as a muscle quality index [22]. Thus, we used RHGS instead of absolute handgrip strength, defined as the average value for maximum grip strength of the dominant hand divided by BMI, which was calculated as weight divided by height squared (kg/m^2^) [25].

### Covariates

Covariates included age, sex, marital status (single, married, divorced, widow), body mass index, physical activity (not active, sedentary, low active, active, very active), depression (defined as self-reported physician diagnosis, medication use), Alzheimer’s (defined as self-reported and medication use) and Parkinson’s (defined as self-reported and medication use). Diabetes was defined as HbA1C ≥ 6.5, and HbA1C level was used to assess whether the diabetes is controlled [26–28].

Hypertension (defined as medication use, systolic blood pressure ≥ 140 mmHg, or diastolic blood pressure ≥ 90 mmHg), current, past smoking (Yes regularly, Yes Occasionally, No), and Glomerular filtration rate (GFR) was calculated by standard formulae.

Each participant’s height was measured using a stadiometer, with a precision of 1 cm. The participant’s weight was measured while wearing light clothing and no shoes, using scales with a precision of 100 g. The body mass index (BMI) was defined as the weight in kilograms divided by height in meters squared (weight (kg)/ [height (m) ^2^).

### Statistical analysis

The normal distribution of the data was checked. The general characteristics of the participants were evaluated for anemic and non-anemic groups. For categorical and continuous data x^2^, and t-test were used, respectively. The relationship between cognitive impairment and hematological parameters was evaluated by logistic regression. Besides this, the linear regression analyses were used to investigate the relationship between physical performances, including mean handgrip strength (muscle strength), Relative Handgrip Strength (RHGS), and 4.57-m usual gait speed, and cognitive function included category fluency test, and late recall with hemoglobin level. Covariates that have a significant clinical and pathophysiological association with desired outcomes of this study (e.g., age, sex, marital status, body mass index, physical activity, depression, diabetes, hypertension, smoking, Alzheimer & Parkinson disease, and GFR) were first assessed by univariate regression models. Covariates that were statistically significant were used in multivariate regression analyses. Then they were used as four regression models to adjust the relationship between hemoglobin, physical performance, and cognitive function. In the first model (model 1), the relationship between anemia, physical performance, and cognitive Function was adjusted with age. in model 2, this relationship was adjusted with age and education level. In the third statistical model (model 3), the adjustment was with age, education level, marital status, BMI, smoking status. In the last statistical model (model 4), HTN, HbA1c, GFR, Alzheimer’s, and Parkinson’s were added to the model 3. Stata MP (version 15) was used, and a *p*-value of < 0.05 was taken as statistically significant for all analyses.

## Results

Of 2426 participants, 1260 (51.94%) were female. The mean age was 69.34 ± 6.39 years. Two hundred twenty-three of the participants (7.43%) were anemic (Table 1). Among participants with anemia, 197 (88.34%) had normocytic anemia (Table 2).

**Table 1:** Characteristics of the participants by anemia status; BEH program (*n* = 2426)

Variable		Total (2426)	Anemic (223)	Non-anemic (2777)	***P***-value
Mean age (years)		69.34 ± 6.39	69.16 ± 6.25	71.07 ± 7.43	< 0.0001
Female sex (%)		1545 (51.50)	108 (48.43)	1340 (48.25)	0.340
Marital status, n (%)	Single	25 (0.83)	–	25 (0.90)	
	Married	2248 (74.93)	167 (74.89)	2081 (74.94)	
	Divorce	26 (0.87)	3 (1.35)	23 (0.83)	
	Widow	701 (23.37)	53 (23.77)	648 (23.33)	
Mean hemoglobin concentration (g/dl) (SD)	Males	15.06 ± 1.73	11.89 ± 0.95	15.41 ± 1.42	0.0001
	Females	13.96 ± 1.56	11.14 ± 0.87	14.22 ± 1.33	0.0001
Mean BMI (kg/m^2^)	Males	25.20 ± 4.01	25.20 ± 4.01	26.35 ± 4.00	0.003
	Females	28.70 ± 5.33	28.26 ± 6.10	28.74 ± 5.26	0.365
Mean hand grip		22.16 ± 9.23	19.88 ± 8.19	22.39 ± 9.29	0.0001
Walking 4.57 m/s		6.68 ± 5.75	7.15 ± 4.03	6.64 ± 5.89	0.203
Positive for Hypertension		1818 (75.16)	178 (79.82)	1640 (74.68)	0.170
Positive for Diabetes		1228 (50.68)	118 (52.91)	1110 (50.45)	0.713
Positive Cognitive disorder, n (%)		1194 (39.80)	115 (51.57)	1079 (38.85)	0.0001
Smoking	None	735 (30.32)	84 (37.67)	651 (29.58)	0.021
	Past cigarette or hookah	1185 (48.89)	104 (46.64)	1081 (49.11)	
	current cigarette or hookah	504 (20.79)	35 (15.70)	469 (21.31)	
Physical activity, n (%)	Not active	155 (6.39)	25 (11.21)	130 (5.91)	0.001
	Sedentary	1714 (70.71)	164 (73.54)	1550 (70.42)	
	Low active	397 (16.38)	22 (9.87)	375 (17.04)	
	Active	132 (5.45)	8 (3.59)	124 (5.63)	
	Very active	26 (1.07)	4 (1.79)	22 (1.00)	

For categorical and continuous data x^2^, and t-test were used respectively

*BEH Program* Bushehr Elderly Health Program, *BMI* The body mass index

**Table 2:** The types of anemia in the BEH program

Types of anemia	Anemia		
	Mild	Moderate to severe	Total
**Microcyte anemia, n (%)**	19 (8.52)	2 (0.90)	21 (9.42)
**Normocyte anemia, n (%)**	**189 (84.75**)	8 (3.59)	197 (88.34)
**Macrocyte anemia, n (%)**	5 (2.24)	0 (0.00)	5 (2.24)
**Total, n (%)**	213 (95.52)	10 (4.48)	223 (100)

*Notes*: Microcyte Anemia was defined as MCV lower than 80 femtolitres (MCV < 80) and normocyte anemia (MCV 80 to 100) femtolitre and macrocyte anemia were define as (MCV > 100) femtolitre

Anemic cases were divided into severe and mild anemia. Mild anemia is defined as HGB (Hb) concentration between 10 to 12 g/dl in women and 10 to 13 g/dl in men. Moderate to severe was determined as Hgb concentration lower than 10 g/dl in both sexes

Table 1 shows the sample characteristics between the anemic and non-anemic groups. For both genders, there was a significant difference between anemic and non-anemic participants in age, marital status, mean BMI, and mean Hgb concentration. Among men, BMI was significantly lower in the anemic group, but not in women. There was a significant difference in physical activity between the non-anemic and anemic groups (*P* = 0.001). Compared to non-anemic participants, those with anemia were more sedentary and not-active; they also had a significantly lower mean handgrip (*P* = 0.0001). Besides, the prevalence of cognitive disorder was significantly higher among anemic participants (51.57%) than those without anemia (38.58%) (*p* = 0.0001).

The associations of hematological parameters with cognition are shown in Table 3. RBC, mean Hgb, and HCT were significantly lower in the participants with cognitive impairment and had preventive effect from cognitive impairment. [OR (95%CI) = 0.7 (0.65 to 0.89), *P*-value = 0.0001 for RBC; OR (95%CI) = 0.94 (0.89 to 0.98), *P*-value = 0.009 for Hgb; OR (95%CI) = 0.96 (0.95 to 0.98), *P*-value = 0.0001 for HCT].

**Table 3:** The relationship between cognitive impairment and hematological parameters in the BEH Program (*n* = 2426)

Variable	Cognitive disorder		OR [95% CI]	***P***-value
	Positive	Negative		
**RBC**	4.96 ± 0.66	5.06 ± 0.61	0.7 (0.65 to 0.89)	**0.0001**
WBC	7.35 ± 1.82	7.39 ± 2.49	0.99 (0.95 to 1.02)	0.637
MCV	85.78 ± 8.44	85.67 ± 8.14	1.00 (0.99 to 1.01)	0.747
MCH	29.73 ± 3.40	29. 88 ± 9.09	0.99 (0.9 to 1.00)	0.56
RDW	16.05 ± 54.73	14.38 ± 3.62	1.01 (0.98 to 1.04)	0.445
RDWa	75.68 ± 23.14	74.90 ± 20.32	1.00 (0.99 to 1.00)	0.401
**Hgb**	14.4 ± 1. 82	14.58 ± 1.65	0.94 (0. 89 to 0.98)	**0.009**
**HCT**	42.45 ± 5.06	43.19 ± 4.69	0.96 (0.95 to 0.98)	**0.0001**

The logistic regression was used for analysis

*BEH Program* Bushehr Elderly Health Program, *CBC* complete blood count, *Hgb* hemoglobin, *MCH* Mean corpuscular hemoglobin, *MCV* Mean corpuscular volume, *PLT* Platelets, *RBC* Red blood cell count, *RDW* Red Cell Distribution Width, *WBC* White blood cells

**Fig 1:**
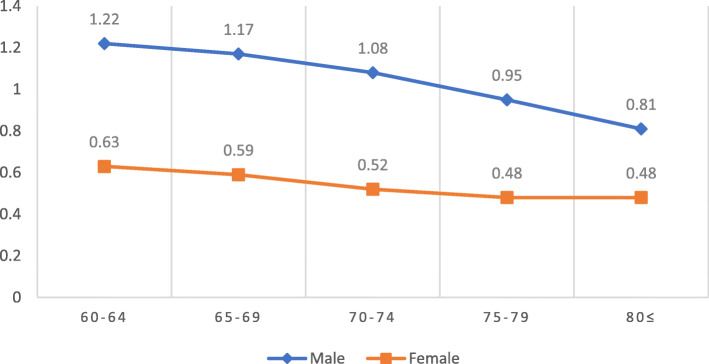
The relative handgrip among male and female based on age category

**Fig. 2:**
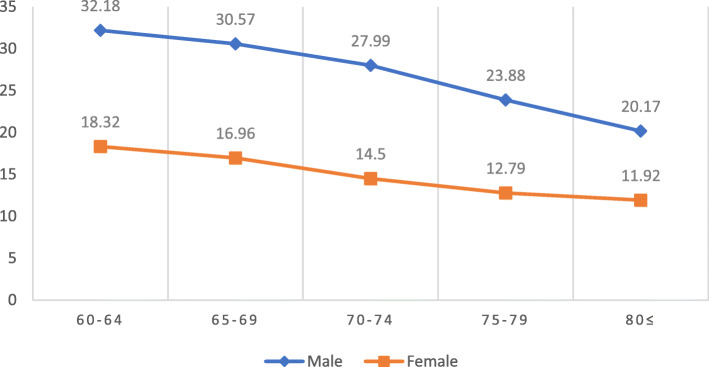
The mean handgrip among male and female based on age category

**Fig. 3:**
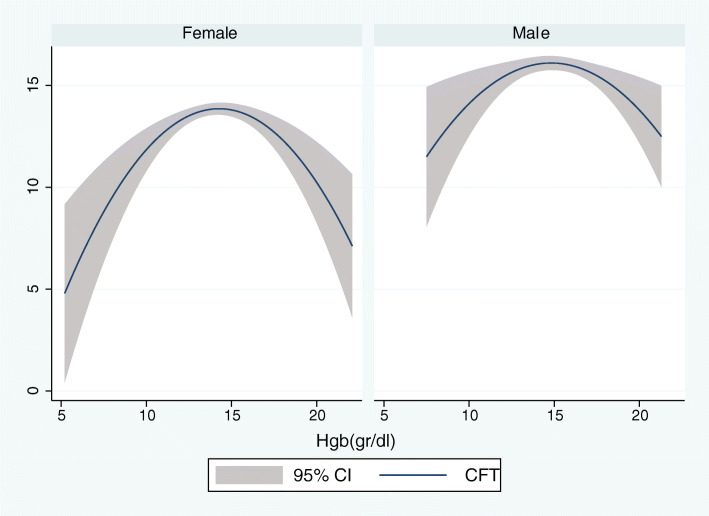
Fractional polynomial plot and 95% CI association between HGB and CFT

**Fig. 4:**
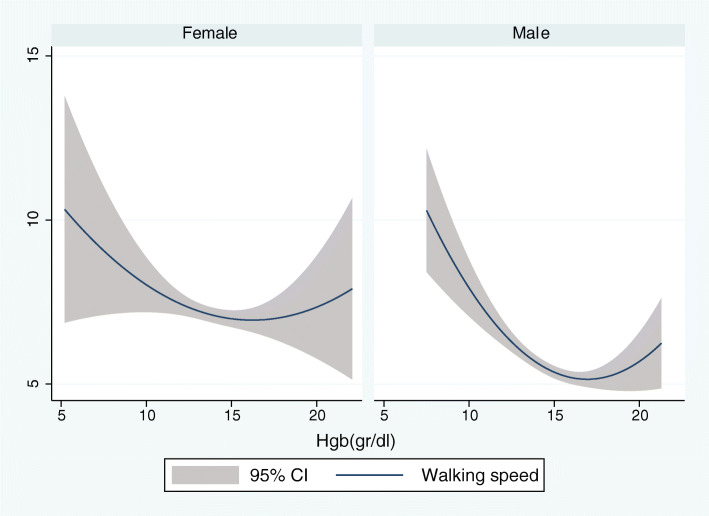
Fractional polynomial plot and 95% CI association between HGB and walking speed

**Fig. 5:**
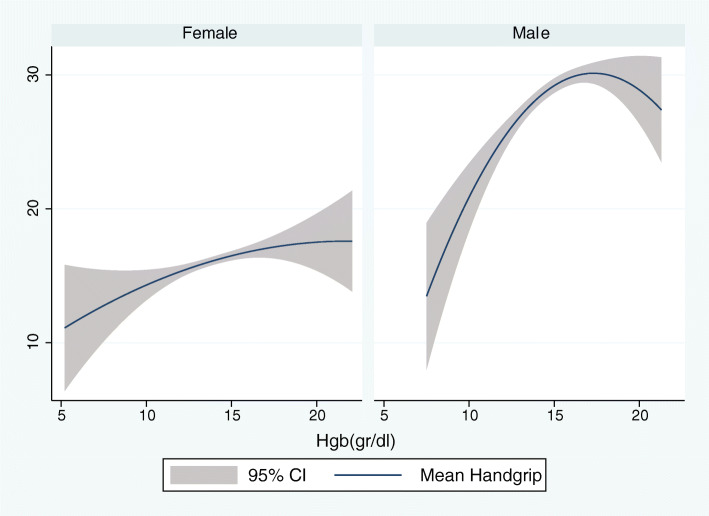
Fractional polynomial plot and 95% CI association between HGB and mean hand grip

According to the results of linear regression shown in Table 4, Hgb concentration was positively associated with the category fluency test, late recall, mean handgrip, relative handgrip, while Hgb concentration had a reverse association with the usual gate speed. After adjustment for education, the association between Hgb concentration and category fluency test was no longer significant [β (95%CI) =0.09 (− 0.01 to 0.19), *P* = 0.081]. In contrast, the association between Hgb concentration, the late recall test, mean handgrip, relative handgrip, and usual gate speed was significant even after education, and other confounders were considered (model 4) [β (95%CI) =0.03 (0.00 to 0.05), *P* = 0.010 for late recall test; β (95%CI) = 1.30 (1.11 to 1.48), *P* = 0. 0001 for mean handgrip; β (95%CI) = 0.04 (0.03 to 0.05), *P* = 0.0001 for relative handgrip; β (95%CI) = − 0.12 (− 0.22 to − 0.01), *P* = 0.020 for usual gate speed]. After stratifying the participants by gender, the association between Hgb concentration, Category fluency test, and late recall was no longer significant (all *p*-value > 0.05). Alternatively, when gender was considered separately, the Hgb concentration association with usual gate speed was decreased significantly in both men and women. As shown in Figs. 1 and 2, age was inversely associated with the mean handgrip and relative handgrip in both men and women, which were more potent in men. These results indicated that Hgb concentration was significantly associated with the mean handgrip and relative handgrip, while usual handgrip, late recall, and Category fluency test (semantic memory) were more affected by gender. Moreover, Figs. 3, 4, and 5 show the relationship between Hgb concentration, CFT, walking speed, and mean handgrip separately for men and women.

**Table 4:** Gender-stratified relationship between cognitive &physical measures and anemia; BEH program (*n* = 2426)

Outcome variable	Analytic model	All		Male		Female	
		β (95% CI)	*P*-value	β (95% CI)	*P*-value	β (95% CI)	*P*-value
Hemoglobin ^a^							
Category fluency test (semantic memory)	Crude	0.20 (0.09 to 0.32)	0.0001	− 0.02 (− 0.19 to 0.14)	0.781	0.03 (− 0.13 to 0.19)	0.725
	Model 1	0.14 (0.02 to 0.25)	0.014	−0.12 (− 0.29 to 0.04)	0.148	− 0.03 (− 0.19 to 0.12)	0.681
	Model 2	0.09 (− 0.01 to 0.19)	0.081	0.00 (− 0.15 to 0.16)	0.922	0.07 (− 0.07 to 0.22)	0.343
	Model 3	0.06 (−0.04 to 0.17)	0.251	−0.01 (− 0.17 to 0.14)	0.838	0.05 (− 0.09 to 0.20)	0.481
	Model 4	0.05 (−0.05 to 0.16)	0.342	−0.07 (− 0.23 to 0.08)	0.365	0.01 (− 0.13 to 0.17)	0. 809
Late recall	Crude	0.02 (0.00 to 0.04)	0.028	0.02 (−0.00 to 0.06)	0.140	0.02 (−0.00 to 0.06)	0.112
	Model 1	0.03 (0.01 to 0.05)	0.003	0.03 (0.00 to 0.07)	0.029	0.03 (0.00 to 0.07)	0.038
	Model 2	0.03 (0.01 to 0.05)	0.003	0.02(−0.01 to 0.05)	0.198	0.02 (−0.00 to 0.06)	0.154
	Model 3	0.03 (0.00 to 0.05)	0.011	0.02 (−0.01 to 0.05)	0.169	0.03 (−0.00 to 0.06)	0.094
	Model 4	0.03 (0.00 to 0.05)	0.010	0.02 (−0.01 to 0.05)	0.185	0.03 (−0.00 to 0.06)	0.080
Mean hand grip	Crude	1.69 (1.49 to 1.89)	0.0001	0.80 (0.53 to 1.07)	0.0001	0.36 (0.18 to 0.53)	0.0001
	Model 1	1.56 (1.36 to 1.75)	0.0001	0.52 (0.28 to 0.76)	0.0001	0.25 (0.09 to 0.42)	0.002
	Model 2	1.50 (1.32to1.68)	0.0001	0.84 (0.58 to 1.10)	0.0001	0.38 (0.21 to 0. 56)	0.0001
	Model 3	1.30 (1.11 to 1.48)	0.0001	0.78 (0.52 to 1.04)	0.0001	0.35 (0.17 to0.52)	0.0001
	Model 4	1.30 (1.11 to 1. 48)	0.0001	0.57 (90.31 to 0.83)	0.0001	0.23 (0.06 to 0. 40)	0.006
Relative hand grip	Crude	0.06 (0.05 to 0.07)	0.0001	0.02 (0.01 to 0.03)	0.0001	0.01 (0.00 to 0.01)	0.001
	Model 1	0.06 (0.05 to 0.06)	0.0001	0.01 (0.00 to 0.02)	0.019	0.00 (0.00 to 0.01)	0.011
	Model 2	0.05 (0.04 to0.06)	0.0001	0.02 (0.01 to 0.03)	0.0001	0.01 (0.00 to 0.01)	0.0001
	Model 3	0.04 (0.03 to 0.05)	0.0001	0.01 (0.00 to 0.01)	0.0001	0.01 (0.00 to 0.01)	0.0001
	Model 4	0.04 (0.03 to 0.05)	0.0001	0.01 (0.00 to 0.02)	0.0001	0.00 (0.00 to 0.01)	0.004
Walking 4.57 m/s	Crude	−0.33 (− 0.46 to − 0.19)	0.0001	− 0.14 (− 0.25 to − 0.03)	0.010	− 0.17 (− 0.43 to 0.08)	0.178
	Model 1	− 0.24 (− 0.36 to − 0.11)	0.0001	−0.06 (− 0.17 to 0.03)	0.021	− 0.05 (− 0.29 to 0.19)	0.686
	Model 2	−0.28 (− 0.41 to-0.14)	0.0001	− 0.15 (− 0.26 to − 0.04)	0.007	−0.20 (− 0.45 to 0.05)	0.118
	Model 3	−0.20(− 0.34 to − 0.07)	0.002	−0.14 (− 0.25 to − 0.03)	0.013	−0.14 (− 0.40 to 0.11)	0.285
	Model 4	−0.12 (− 0.22 to − 0.01)	0.020	−0.04(− 0.16 to 0.06)	0.376	0.04 (− 0.13 to 0.22)	0.589

The linear regression was used for analysis

a Hemoglobin concentration was used as an independent variable

Model 1 adjusted for age

Model 2 adjusted for education

Model 3 adjusted for age, education level, marital status, BMI, smoking status

Model 4 adjusted for age, education level, marital status, BMI, smoking status, HTN, HbA1c, GFR,Alzehimer’s, Parkinson’s

## Discussion

In the present study, we found a significant relationship between anemia and physical function variables such as mean handgrip, relative handgrip, and walking speed. Furthermore, there was an association between anemia and cognitive function variables such as semantic memory and late recall after adjusting for different demographic and clinical variables. We found an association between anemia and walking speed only among men in gender-stratified analyses. Besides, we did not find an association between anemia and cognitive function in gender-stratified evaluations.

Our analyses indicated that approximately 7.43% of the participants were anemic. Various studies estimated the prevalence of anemia in the elderly, with different conditions and characteristics. Anemia prevalence was 19% in the Iranian elderly study in Amir Cola [29].14.6% of older adults had anemia in an Australian epidemiologic study [30]. Moreover, in a systematic review, including 34 studies, the prevalence of anemia was reported 17% [31]. The contrast in the prevalence of anemia might be due to the diagnostic criteria for anemia, heterogeneity of participants in the race, living conditions, and health problems. Besides, the lower average age of the participants and the higher prevalence of smokers may also contribute to the difference between anemia prevalence in our study with others. The result of our study was consistent with previous cross-sectional and longitudinal studies, which exhibited the prevalence of anemia increased with advancing age [5, 29, 31, 32]. An increase in Hgb concentration in smokers could be caused by carbon monoxide exposure, which is more likely to reduce the prevalence of anemia among smokers. Some studies investigated that increasing Hgb levels in smokers might be a compensatory mechanism. Carbon monoxide (CO) binds to hemoglobin with greater affinity than oxygen. After smoking, carbon-monoxide-hemoglobin causes the kidney to detect less oxygen, hypoxia, thus producing more erythrocytes. It affects hemoglobin levels [33]. Therefore, smokers have higher Hgb levels than non-smokers, which is more likely to reduce the prevalence of anemia among smokers. According to previous studies, Hookah smoke is more harmful than cigarettes for passive smokers [34]. It causes an increase in Hgb concentration. The elderly in this study prefer to smoke hookah due to their culture. Therefore, higher Hgb levels in this group are acceptable.

In this study, the most common type of anemia was normocytic. The advanced aged often suffer from chronic diseases, which explains the high prevalence of normocytic anemia in the elderly. This finding was similar to previous studies about the elderly [32].

There was a significant association between hemoglobin levels, RHGS, and handgrip. The association was still substantial and significant after adjusting for potential confounders and considering gender separately. Other studies such as Yu-mi Gi et al. (2020) in Korea [35] and Santos et al. (2018) in Brazil [36] have confirmed a significant relationship between hand-grip and anemia. The mechanisms for the association between Hgb concentration and muscle strength decline are not fully understood. Hgb plays an essential role in the oxygenation of the muscle tissue. As a result, anemia is associated with a decrease in muscle strength. Anemia decreases the oxygen-carrying capacity, resulting in tissue hypoxia and leads to poor outcomes such as failing muscle strength.

There are some compensatory mechanisms such as dilation of peripheral arteries, activation of the sympathetic angiotensin, and renin aldosterone systems, which cause to maintain blood pressure.

A long-term increase in cardiac output causes left ventricular hypertrophy. That might lead to cardiovascular disease, especially congestive heart failure, which is one of the most common causes of disability [35].

In older individuals, various tissue functions are decreased due to frailty, aging, and chronic diseases. Therefore, anemia might cause tachypnea, tachycardia, decreased exercise tolerance, muscle mass loss, and decreased physical performance. According to some studies, anemia may share a pathophysiological pathway with chronic inflammatory processes that might explain the relationship between anemia and physical function deterioration [35, 37]. In contrast to our research, Joosten et al. (2016) [38] found no association between anemia and physical performance variables such as handgrip and usual gait speed among hospitalized elderly. The differences may be due to the age of participants, their fragility as well as their acute stage, and being hospitalized. The association between anemia and handgrip was in line with previous literature demonstrating that age increasing is related to decreased hemoglobin levels.

In Asian and Iranian culture, particularly in older individuals, women have more responsibility than men in doing housework such as cooking, cleaning, washing dishes, taking care of (grand) children, and their husbands. Therefore, they could maintain muscle strength, especially in their hands. Several previous studies indicated that older men have a higher chance of losing muscle mass with increasing age than older women. This mechanism is not understood completely [35].

Decreases in physiological factors (e.g., insulin-like growth factor-1 and testosterone) and social factors such as work retirement and loss of social roles might dramatically decrease muscle strength in older men, affecting handgrip and their daily activities.

Older adults might have high levels of inflammatory factors due to their underlying chronic diseases. Acute and chronic inflammatory processes decrease Hgb levels and physical function.

At an older age, anemia of chronic diseases is more frequent than age-related anemia. This study found an independent relationship between Hgb levels and physical and cognitive outcomes after adjusting for some chronic diseases related to anemia, such as diabetes, hypertension, and kidney disease.

In some studies, an association between anemia and physical function was still significant after adjusting for underlying diseases [35, 39]. However, with the increase in the number of these diseases, the severity of the relationship has decreased. Maraldy et al.’s (2006) [40] study was in contrast to this result.

As confirmed in this study, anemic individuals are more inactive and have sedentary lifestyles, which might affect physical performance. Besides, physical activity increases red blood cell production and oxygen-carrying capacity. Older people with a higher level of Hgb are more physically active; therefore, they have higher muscle mass and strength. One of the most common symptoms of anemia is fatigue. It can significantly limit physical activity, leading to decreased muscle mass and strength [35].

There was a significant difference between the physical activities in anemic and non-anemic participants in the present study. However, according to the adapted model, the relationship between anemia and physical function outcomes, e.g., handgrip, RHGS, usual gait speed, was independent of the effect of physical activity.

Interestingly, our result shows that the association between Hgb concentration and usual gait speed was no longer statistically significant in women when participants were stratified by gender, which indicated a gender-related effect on usual gait speed. This result is consistent with previous studies that demonstrated the influences of age and gender on the usual gait speed [41–45]. As studies showed, stride-length and step-length, which are relevant factors for gait speed, were higher in men [46–48]. This result may explain by differences in body composition and size. Notably, gait speed is also affected by culture, lifestyle, and socio-demographics such as education, occupational class, and income. For instance, less educated elderly have lower gait speed and physical activity due to a higher prevalence of chronic underlying diseases, smoking, and obesity [41]. Additionally, studies showed that the level of education is a stronger predictor in men than women. In this study, men were found to have a significantly higher level of education than women.

As this study shows, after considering gender separately, the association between Hgb concentration and cognitive parameters was no longer significant. This finding indicates the gender differences in cognitive function, consistent with previous studies [49–53]. Additionally, women had a lower cognitive function than men in this study. A possible explanation for this result is differences in the sex hormones changes. As mentioned in the studies, sex hormones have a protective role against cognitive function decline [50, 54, 55]. Furthermore, post-menopausal women have a significant decline in sex hormones, making them more susceptible to cognitive impairment [56, 57].. Another possible explanation for gender differences in cognitive function is education level disparities. In this study, women had a lower level of education, and when we consider education into account for the whole participants, the association between Hgb concentration and semantic memory was no longer significant. This finding supports previous research that confirms the effect of years of formal education on cognitive reserve as a predictor of cognitive impairment in the elderly [58–62]. Moreover, in developing countries such as Iran, women have a lower chance of participation in cognitively demanding jobs, cognitive leisure activities (e.g., reading books, doing word games or puzzles), and intellectual activities due to low education levels and socioeconomic status, which make women more susceptible to cognitive impairment than men [60, 63–66].

Our finding provides a contrast with some previous studies that confirmed the relation between anemia and cognitive impairment [32, 52, 67–71]. These differences might be due to the relatively younger age of participants in this study. Furthermore, it seems that the elderly with older age, less education, and underlying disease are less likely to participate in the studies due to lower physical and cognitive function. Individuals with a lower level of hemoglobin would be unlikely to survive to participate in the studies [13]. Adjusting for multi-confounders might also affect the relationship between Hgb and cognitive impairment by causing overmatching bias, which reduces the study precision and might obfuscate the relation between variables [46, 72]. Additionally, the cognitive function would likely be affected by culture, education level, and socioeconomic factors more than physical function, which is objective [73, 74].

Regardless of the effect of gender on the association between anemia and cognitive impairment, mechanisms explaining Hgb change, which have a pronounced effect on women than men, are not fully understood [7]. Further longitudinal studies with higher mean age are required to establish the effect of gender and being women on the association between Hgb concentration and cognitive function.

Our analysis has several strengths; first, a large number of participants represents a sample of a community of older adults of both genders in Iran. Second, a comprehensive measurement of demographic and health-related confounders allows us to explore the association between Hgb concentration and physical and cognitive function. Second, previous studies in this field have generally been conducted in high-income countries (HICs) and developed countries. Lower and middle-income countries (LMICs) such as the Middle East countries have a smaller share of these studies. This population-based study was performed using reliable data and a fully validated protocol. Besides, we examined the effect of anemia on both cognitive and physical function simultaneously as outcome variables in this study, considering various covariates such as sociodemographic factors, lifestyle factors, and illness-related factors. Moreover, the relationship between these variables has still remained controversial despite several studies in this field detailed in Table 5. In this study, we try to clarify this relationship by conducting a study on large community-dwelling elderly. Finally, the physical activity assessment was carried out via objective and questionnaire methods simultaneously; moreover, two different cognitive domains, semantic and late recall, were measured, which are an important component of the cognitive function.

**Table 5:** Characteristics of studies worked on the relationship between anemia, cognitive and physical function

Author	Population baseline numbers descriptive Length of follow up	Type of study main outcome	Measurements Of cognition or hand grip	Measurements Of anemia	Adjusted for:	Relationship between anemia and cognitive	Statistics
Trevisa-n et al 2016 [53]	e Progetto Veneto Anziani project on, Italian population, 1227 participants older than 65 years old, without cognitive impairment mean follow up 4.4 (1.2SD) years	Cohort the onset of the cognitive impairment	the 30-item Mini-Mental State Examination (MMSE)	Based on WHO criteria Samples were Divided into the gender-specific Hb tertiles using the following cut-offs: 13.9 and 14.9 g/dL for men; and 12.8 and 13.7 g/dL for women.	Age, sex, education, smoking, alcohol, monthly income, living alone, physical activity, BMI, hearing loss, vision loss, hypertension, CVD, COPD, OAD, diabetes, cancer	Low hg concentration Increases the risk of cognitive impairment in the elderly, apparently with a stronger association in men than in women.	Participants with the lowest Hb concentrations had a significant 37% higher risk (95% confidence interval [CI]: 1.08–1.75; *p* = 0.01) of being diagnosed with cognitive impairment. Considering the gender separately, the risk of cognitive impairment only increased significantly, by 60%, for men in the lowest Hb tertile (95% CI: 1.06–2.41; *p* = 0.02), but not for women (hazard ratio = 1.32; 95% CI: 0.97–1.79; *p* = 0.08).
Dlugaja et al 2015 [69]	4033 participants from mandatory city registries in the Ruhr area in Germany, participant 45 to 75 years of age Five years follow up	Cohort Anemia and mild cognitive impairment	verbal memory measured by a word list consisting of eight words from the Nuremberg Geriatric Inventory Speed of processing/executive functioning was measured using the labyrinth test, a paper-pencil test from the NAI For mild cognitive impairment diagnosis: Participants were asked if their cognitive performance changed during the past two years, then statistical manual of mental disorders,	WHO criteria hemoglobin level < 13 g/dl in men and < 12 g/dl in women	Age, gender, BMI, education, diabetes, blood pressure hypertension, stroke, cancer, depression scale, smoking status, total cholesterol	Anemic participants showed lower performances in verbal memory and executive functions	Adjusted Odds ratios (OR) for mild cognitive impairment (MCI), amnestic- MCI, and non-amnestic-MCI in anemic versus non-anemic participants were 1.92 (95%-CI, 1.09–3.39), 1.96 (1.00–3.87), and 1.88 (0.91–3.87).
Payne et al 2018 [13]	4499 men and women aged 40 and over	cross-sectional data from a population-based study of rural South African men and women physical and cognitive performance	Grip strength was measured twice in both hands, using a Smedley digital dynamometer (12–0286). Walk speed was measured by asking participants to walk a 2.5 m course twice, with the time taken timed to the nearest 0.1 s. Cognitive performance was assessed with a cognitive battery adapted for language, cultural, and educational appropriateness from validated measures used in the U.S. Health and Retirement Study.	Hg concentration < 12 g/dL for women and < 13 g/dL for men	Age, sex, education, Median C reactive protein concentration, HIV, hypertension, diabetes mellitus, mean body mass index, and self-reported angina, chronic bronchitis, and stroke	There was no association between hemoglobin levels and walk speed or cognitive score Hemoglobin concentration Was independently associated With grip strength	Hemoglobin concentration Was independently associated With grip strength in women when covariates were included in the model (B = 0.391; 95% CI 0.177 to 0.605), but this association was not statistically significant in men (B = 0.266; 95% CI − 0.019 to 0.552
Qin et al 2019 [75]	9324 adults aged 45 years or older from the China Health and Retirement Longitudinal Study	Cohort Association between Anemia and cognitive decline among Chinese middle-aged and elderly	Cognitive performance assessed by memory recall (episodic memory), mental status (TICS), and global cognitive function at baseline survey	WHO criteria hemoglobin level < 13 g/dl in men and < 12 g/dl in women	Age, gender, education, marital status, cigarette, smoking, body mass index, hypertension, diabetes, abdominal adiposity, chronic pain, dyslipidemia, CRP, HDL, and cholesterol	This study found a cross-sectional and longitudinal association between Anemia and accelerated decline in cognitive functions in Chinese middle-aged and elderly The hemoglobin concentration Was associated with global cognitive function global -cognitive function and episodic- memory was associated with anemia independent of covariates	After adjusting for socio-demographic and health-related covariates, the cross-sectional association between anemia and global cognitive function [β (95%CI) = − 0.49(− 0.69 ~ − 0.29)], episodic memory [β (95%CI) = − 0.14(− 0.23 ~ − 0.05)], and TICS [β (95%CI) = − 0.23(− 0.38 ~ − 0.08)] were significant and did not differ by gender.
Joosten Et al 2016 [38]	220 patients aged 70 years and older	Prospective study The relationship Between Anemia and handgrip and walking speed	Handgrip strength was assessed with a hydraulic hand dynamometer. Gait speed (in meters per second) was calculated after a 4.5 m walk ADL score	WHO criteria hemoglobin level < 13 g/dl in men and < 12 g/dl in women	Sex, age, BMI, ADL, CRP, GFR, MMSE mean, cancer, gastrointestinal diseases, −Neuropsychiatric diseases, Falls-fractures-osteoporosis	Handgrip, ADL score, and gait speed were not significantly different in anemic and non-anemic person	No significant correlation was found between the hemoglobin values and the hand-grip strength (Spearman’s rho 0.112, *p* = 0.1) and walking speed (Spearman’s rho 0.04, *p* = 0.69)
Hong- bae et al 2019 [70].	16 observational studies, including eight case-control studies and eight cohort studies, were included in the final analysis In total, 16,765 cognitive impairment cases were surveyed in the meta-analysis.	Meta-Analyzed Studies reporting a relationship between Anemia and cognitive impairment from 1964 to July 10, 2019	cognitive impairment in four articles was diagnosed using a cut-off score of 24 on the MMSE, and one article used the International Working Group (IWS) criteria	anemia was defined according to the WHO criteria of hemoglobin level < 13 g/dl in men and < 12 g/dl in women	gender, mean age, duration of follow-up in cohort studies, number of participants, methodological quality, and studies that adjusted for education, cardiovascular risks, smoking status, apolipoprotein E carrier status, alcohol consumption, and physical activity were used in Sub-group meta-analyses	According to this meta-analyzed, There is a relationship between Anemia and cognitive impairment	Anemia was significantly linked to cognitive impairment (OR or RR 1.51; 95% CI: 1.32–1.73) in a random-effects meta-analysis
Valladã-o Júnior et al 2020 [72]	13,624 participants (mean ages = 51.6 years±9)	Cross-sectional study base on ELSA-Brazil Cohort	scores for verbal learning, late recall, word recognition, a semantic verbal fluency test, and the Trail-Making Test, Part B (TMT-B)	WHO criteria hemoglobin level < 13 g/dl in men and < 12 g/dl in women	Education, race, monthly family income, excessive alcohol use, thyroid function, smoking status, hypertension, diabetes, dyslipidemia, body mass index, Antipsychotic, antiparkinsonian, or anticonvulsant drug use	Hemoglobin levels were not associated with global cognitive scores,	Global cognitive scores were similar between participants with and without anemia in adjusted models for the entire sample (b = − 0.004; 95% CI = –0.052, 0.044) or separately, for men (b = 0.047; 95% CI = –0.053, 0.146) and women (b = − 0.015; 95% CI = –0.070, 0.040)
Jiang et al 2020 [71]	4838 participants 65 years old and over	Cross-sectional	CognitivefunctionwasevaluatedusingtheMini-MentalStateExamination (MMSE) and neuropsychological test battery	WHO criteria hemoglobin level < 13 g/dl in men and < 12 g/dl in women	Demographic factors, lifestyle, and clinical condition	Anemia was associated with cognitive performance There was no relationship between Anemia and dementia	Anemia was associated with a multiple-adjusted odds ratio of 1.28 (95%CI:1.041.57) for MCI and 1.27 (95% CI: 0.87–1.85) for dementia, and a multiple-adjusted β coefficient of − 0.60 (95% CI: − 0.94 to − 0.27) for MMSE score
Brenda et al 2004 [37]	1156 participants aged 65 and older from CHIANTI Study (Italy)	Used data from the Italian National Research Council of Aging	ADL (6 item questioner) IADL (8 items) Walking speed: was defined as the best performance (time in seconds) of two 4-m walks. Standing balance: participants were asked to stand with the feet side by side, a semi tandem position, and a full-tandem position. Chair stand test: participants were asked to stand up from and sit down in a chair five times without using hands	World Health Organization (WHO) criteria hemoglobin level < 13 g/dl in men and < 12 g/dl in women	Age, sex, BMI, smoke, MMSE, diabetes mellitus, myocardial infection, Angina pectoris, Peripheral artery disease, Congestive heart failure, stroke, cancer, lung disease, Gastric ulcer, Hospitalization in the past year, Creatinine, mg/dL	Anemia is associated with disability, poorer physical performance, and lower muscle strength	anemic persons had more disabilities (1.71 vs 1.04, *P* = 0.002) and poorer performance (8.8 vs 9.6, *P* = 0.003), than persons without Anemia. Anemic persons also had significantly lower knee extensor strength (14.1vs 15.2 kg, *P* = 0.02) and lower handgrip strength (25.3vs27.1 kg, *P* = 0.04) than persons without Anemia
Hirani et al 2016 [39]	1705 Australian men aged 70 years old and over from the Concord Health and Ageing in Men Project Five years follow up	Cross-sectional study, The relationship between HG levels, and sarcopenia, low muscle strength, functional and activities of daily living (ADL), and instrumental ADL (IADL) disabilities in older	muscle strength was assessed by handgrip and participate divided in grip strength less than 26.0 kg versus grip strength 26.0 kg and more. Walking speed: was measure 4 mm speed. Participants with a walking speed of 0.8 m/s or less were classified as having low walking speed. ADL: was assessed by seven items from a modified version of the Katz ADL scale IADL: asks participants how much help they need to perform ten tasks considered necessary for independent living	World Health Organization (WHO) criteria Hb level less than 13 g/dL in men	age, income, body mass index, measures of health, estimated glomerular function, inflammatory markers, and medication use	Low hemoglobin concentration over time is associated with poor functional performance for every 1 g/dL increase in Hb, there was a significant reduction in risk of sarcopenia, slow walking speed, poor grip strength, inability to perform chair stands, and ADL and IADL disabilities	there was a association between Hb and grip strength β coefficient = 1.52, 95% CI = 1.27, 1.78 for unadjusted β coefficient = 1.05, 95% CI = 0.80, 1.30 for age-adjusted and β coefficient = 0.82; 95% CI = 0.55, 1.08, for multivariate-adjusted for walking speed: β coefficient = 0.03, 95% CI = 0.02, 0.03; for unadjusted β coefficient = 0.01, 95% CI = 0.01, 0.02; for age-adjusted and β coefficient = 0.01, 95% CI = 0.004, 0.02,, for multivariate-adjusted.
Thein et al 2009 [45]	328 participants 65 years and older	Cross-sectional study To determine the relationship between HG concentration and functional status, depression, disability, and physical strength, independent of chronic disease	IADL (consists of 13 questions) Handgrip strength: with a handheld dynamometer (in kg), using the mean value after performing the task three times	WHO criteria hemoglobin level < 13 g/dl in men and < 12 g/dl in women	Age, sex, diabetes, hypertension, chronic inflammation, or rheumatoid arthritis	There was a significant association of Anemia with declines in health-related quality of life, functional status, and physical strength	Anemia was associated with greater fatigue (*P* < 0.001), lower handgrip strength (*P* = 0.014), and increased number of disabilities (*P* = 0.005)

This study has a number of potential limitations; first, the cross-sectional nature of this study could not establish cause-effect relationships between Hgb concentration, cognitive function, and physical function. Second, in this study, only memory and verbal aspects of cognitive function were assessed due to the low educational levels of the participants. In contrast, other cognitive contents, e.g., executive speed, attention, and processing function, which might be affected more than the assessed domains by hemoglobin changes, were not evaluated. Second, due to the population-based nature of the study, brain diseases (e.g., Alzheimer’s disease and Parkinson’s) were only self-reported, and specific diagnostic tests were not performed to evaluate these diseases. Finally, confounding variables such as inflammatory factors (e.g., C-reactive-protein) and nutritional-status (e.g., albumin) were not assessed in this study.

In conclusion, our study demonstrated that anemia is strongly associated with physical function in the Iranian elderly population, whereas its association with cognitive function was not statistically significant. This research raises the question of whether hemoglobin level correction improves cognitive and physical function. Further longitudinal research is required to investigate the etiology and consequences of anemia in the elderly. Despite uncertain mechanism to explain how anemia affects physical performance and cognitive function, according to the results of this study and previous ones, it seems hemoglobin screening not only might be effective in diagnosing the cognitive function and physical activity decline in the early stage but also might play an important role in preventing these conditions. Moreover, the handgrip strength is one of the evaluation indicators of frailty Syndrome. In this study, its strong relationship with anemia has been confirmed; hence, this finding can strengthen the hypothesis of a relationship between anemia and frailty. We suggest that screening and timely treatment of anemia in the elderly should be considered as a strategy to reduce frailty and improve the rehabilitation process. We believe an effective strategy for controlling anemia in older adults might improve function, quality of life, and even lifespan. Designing an interventional healthcare system for managing anemia plays an essential role in this strategy.

## Data Availability

A large amount of data has been collected for this study. The datasets on which the conclusions of this manuscript rely are available. Interested researchers may access the data via corresponding author AO (a.ostovar@bpums.ac.ir) or IN (inabipour@gmail.com).

## Abbreviations

BEH Program: Bushehr Elderly Health Program
BMI: The body mass index
CBC: complete blood count
CFT: Category fluency test
CI: Cognitive impairment
CO: Carbon monoxide
GFR: Glomerular function rate;
Hgb: Hemoglobin
MCH: Mean corpuscular hemoglobin
MCV: Mean corpuscular volume
PLT: Platelets
RBC: Red blood cell count
RDW: Red Cell Distribution Width
RHGS: Relative Handgrip Strength
WBC: White blood cells
WHO: World Health Organization
